# Contextual Factors Affecting Implementation of In-hospital Pediatric CPR Quality Improvement Interventions in a Resuscitation Collaborative

**DOI:** 10.1097/pq9.0000000000000455

**Published:** 2021-08-26

**Authors:** Maya Dewan, Allison Parsons, Ken Tegtmeyer, Jesse Wenger, Dana Niles, Tia Raymond, Adam Cheng, Sophie Skellett, Joan Roberts, Priti Jani, Vinay Nadkarni, Heather Wolfe

**Affiliations:** From the * Department of Pediatrics, University of Cincinnati College of Medicine; Cincinnati, Ohio; † Division of Critical Care Medicine, Cincinnati Children’s Hospital Medical Center; Cincinnati, Ohio; ‡ Division of Biomedical Informatics, Cincinnati Children’s Hospital Medical Center; Cincinnati, Ohio; § Division of Critical Care Medicine, Seattle Children’s Hospital, Seattle, Wash.; ¶ The Children’s Hospital of Philadelphia, Philadelphia, Pa.; ∥ Medical City Children’s Hospital, Department of Pediatrics, Division of Cardiac Critical Care, Dallas, Tex.; ** Departments of Pediatrics and Emergency Medicine, Cumming School of Medicine, University of Calgary, Calgary, Canada; †† Department of Paediatric Intensive Care, Great Ormond Street Hospital for Children NHS Foundation Trust, London, United Kingdom; ‡‡ Department of Pediatrics, Section of Critical Care Medicine, The University of Chicago, Comer Children’s Hospital; Chicago, Ill.

## Abstract

**Introduction:**

Pediatric quality improvement (QI) collaboratives are multisite clinical networks that support cooperative learning. Our goal is to identify the contextual facilitators and barriers to implementing QI resuscitation interventions within a multicenter resuscitation collaborative.

**Methods:**

A mixed-methods evaluation of the contextual facilitators and barriers to implementation of a resuscitation QI bundle. We administered a quantitative questionnaire, the Model for Understanding Success in Quality (MUSIQ), to the Pediatric Resuscitation Quality (pediRES-Q) Collaborative. Its primary goal is to optimize the care of children who experience in-hospital cardiac arrest through a resuscitation QI bundle. We also conducted semistructured phone interviews with site primary investigators adapted from the Consolidated Framework for Implementation Research qualitative interview guide.

**Results:**

All 13 actively participating US sites completed the MUSIQ questionnaire. Total MUSIQ scores ranged from 86.0 to 140.5 (median of 118.7, interquartile range 103.6–124.5). Evaluation of the QI team subsection noted a mean score of 5.5 for low implementers and 6.1 for high implementers (*P* = 0.02). We conducted 8 interviews with the local QI team leadership. Contextual facilitators included a unified institutional approach to QI, a fail forward climate, leadership support, strong microculture, knowledge of other organizations, and prioritization of goals. Contextual barriers included low team tenure, no specific allocation of resources, lack of formalized QI training, and lack of support and buy-in by leaders and staff.

**Conclusions:**

Using mixed methods, we identified an association between the local QI team’s strength and the successful implementation of the QI interventions.

## Abstract 2

Supplemental Digital Content is available in the text.

## INTRODUCTION

Pediatric quality improvement (QI) collaboratives are multisite clinical networks that support cooperative learning through shared data analysis, testing changes to improve quality, and sharing collective experiences to advance patient outcomes.^1^ These collaboratives can lead to significant improvements in the care processes and clinical outcomes for children.^1–3^ One such collaborative is the Pediatric Resuscitation Quality (pediRES-Q) Collaborative (ClinicalTrials.gov: NCT02708134), a large, multicenter international pediatric resuscitation QI network established in 2016. The primary goal of the collaborative is to optimize the care of children who experience in-hospital cardiac arrest through the implementation and validation of a resuscitation QI bundle. In-hospital cardiac arrest occurs in more than 7,000 pediatric patients each year.^4^ Many children who require cardiopulmonary resuscitation (CPR) die during or shortly after the event, and survivors may experience new disabilities.^5^ Although substantial progress has reduced the number of in-hospital pediatric cardiac arrest events and improved outcomes for survivors,^6,7^ a critical gap in the implementation of resuscitation best practices remains.^8^ Until reliable implementation of QI interventions is achieved, we will lack a complete understanding of their combined and sustained impact on CPR quality during in-hospital cardiac arrest.

The goal of the pediRES-Q collaborative is to address critical gaps in resuscitation best practice implementation. Despite access to the same QI bundle interventions, reliable implementation of interventions is lacking across centers, and CPR performance has varied across the network.^8^ This is not a unique problem for our collaborative. Within QI initiatives and collaboratives, not all groups perform equally well,^9–11^ and differences in implementation and performance are hypothesized to be due to contextual factors.^12^ Research into contextual factors can aid in implementing QI interventions by identifying contextual facilitators and barriers. Critical contextual factors, including the external environment, structural characteristics, resources, culture, and leadership, can all affect the success of the QI interventions.^13^ By assessing these contextual factors, we hoped to elucidate the facilitators and barriers specific to a particular site and clinical area.^12,14,15^ We hypothesized that QI bundle implementation variability within the pediRES-Q collaborative was due to differences in contextual facilitators and barriers at individual institutions identified by local pediRES-Q collaborative leaders. We, therefore, conducted a mixed-methods study utilizing: (1) quantitative results of a commonly used tool to assess local context; (2) qualitative semistructured interview data from site leaders and their team if available; and (3) compliance with the recommended resuscitation QI bundle. We sought to identify the contextual facilitators and barriers to implementing evidence-based pediatric resuscitation QI interventions as recommended by the pediRes-Q collaborative.

## METHODS

This study was a mixed-methods evaluation of the contextual facilitators and barriers associated with the pediRES-Q recommended resuscitation QI bundle. We selected a mixed-methods approach first to identify contextual weaknesses at a specific site quantitatively and then develop a more detailed understanding of contextual facilitators and barriers to implementation via a qualitative approach. Both the quantitative and qualitative components were necessary to increase understanding and develop strategies to improve implementation. We included all actively participating US sites, defined as participating in the collaborative for at least 12 months and having enrolled at least five patients at the time of study initiation, July 2018. Each site completed the quantitative tool first before the invitation for qualitative interviews. This study was determined to be nonhuman subjects research by the Institutional Review Board at Cincinnati Children’s Hospital Medical Center.

### Resuscitation QI Bundle

The pediRES-Q Collaborative offers a bundle of QI interventions geared toward improving CPR outcomes for children that hospitals may choose to fully or partially implement. The bundle elements include as follows: (1) a checklist (**see Supplemental Digital Content 1,** http://links.lww.com/PQ9/A294) for the identification of patients at risk for cardiac arrest^16,17^; (2) rolling refreshers to provide bedside just-in-time CPR training^18^; (3) structured “hot” debriefings immediately following cardiac arrest events^19^; (4) “cold” data-informed cardiac arrest debriefings provided at a later time^20,21^; and (5) a postcardiac arrest care checklist (**see Supplemental Digital Content 1,** http://links.lww.com/PQ9/A294). We limited our evaluation to determine the association between local contextual factors and compliance with rolling refreshers, hot debriefing, and cold debriefing (**see Appendix I, Supplemental Digital Content 2,** http://links.lww.com/PQ9/A295 for further description) due to their established evidence base.

### Compliance Data

For each cardiac arrest, QI bundle elements are entered into the deidentified central database maintained for quality by the pediRES-Q collaborative staff.

### Study Definitions

We defined low implementers as those sites successfully implementing 0–1 of the 3 QI interventions. We divided low implementers into two subgroups for the qualitative interviews: those attempting implementation of multiple QI bundle elements, referred to as “distributed approach low implementers,” and those with a focused approach resulting in a highly reliable (>90%) implementation of only one bundle element referred to as “focused approach low implementers.” We defined high implementers as sites implementing 2–3 interventions successfully.

Successful implementation was defined a priori using predetermined criteria derived by group-consensus of the manuscript authors and agreed upon by collaborative leadership. We defined successful implementation of CPR rolling refreshers as completing a rolling refresher on at least 50% of patients identified as high risk. We defined hot debriefing implementation as completing a hot debrief for at least 50% of in-hospital cardiac arrests. We defined cold debriefing based on the number of yearly events due to no clear recommendation on the optimal completion of cold debriefs and the significant time required to complete them. We defined successful cold debriefing implementation as debriefing 50% of events for those centers who had <10 in-hospital cardiac arrests per year and as at least 10 debriefs over the year for those centers having more than 10 in-hospital cardiac arrests per year. Although these predetermined compliance levels are below thresholds normally used for high reliability,^22^ the study team chose them due to the infrequent levels of debriefing implemented in prior research studies.^19,23,24^

### Quantitative Data Collection

We administered an Excel-based, quantitative tool to assess local context, the Model for Understanding Success in Quality (MUSIQ),^14^ to all 13 actively participating US sites in the pediRES-Q Collaborative over 1 month. We chose the MUSIQ framework, which is the most popular contextual framework, and a questionnaire adapted from the framework as our measurement tool. This tool is not extensively validated, but its face validity is well established, and its criterion validity is documented in an exploratory analysis of 74 projects.^25^ MUSIQ identifies 24 contextual factors mapping to 6 domains that may influence QI success: external environment, organization, QI support and capacity, QI team, microsystem, and miscellaneous. The survey tool (https://qi.elft.nhs.uk/resource/the-model-for-understanding-success-in-quality-2/) contains 37 questions with a score range from 24 to 168. Total score assessment developed by expert consensus (per personal communication with MUSIQ developer Lloyd Provost) outlines a score of 120–168 indicating that a project has a reasonable chance of success, a score of 80–119 indicating possible contextual barriers, and a score of 50–79 indicating serious contextual issues and concerns for success. Each contextual factor is measured on a 7-point Likert scale, and most contextual factors are assessed with a single question. Contextual factors within microsystems and those related to the QI team directly shape QI success, whereas factors within the organization and external environment indirectly influence success.^14^ Site primary investigators completed the survey over one month. As our focus was on the contextual facilitators and barriers to implementation as experienced by local leaders, the MUSIQ tool was sent to the collaborative team leader. A physician fulfilled this role for all 13 sites.

### Quantitative Data Analysis

We completed summary statistics with counts, proportions (%), mean, median, interquartile range (IQR), and SD as appropriate. We evaluated differences between total scores by high and low implementers via Wilcoxon Rank Sum. We compared institutions within total score categories via Chi-Square and differences explored in the MUSIQ tool subsection scores by high and low implementer sites. We calculated differences between institutions using the two-tailed t-tests, and *P* values less than 0.05 were considered statistically significant. Based upon an estimated difference in MUSIQ subsection score of 1 with an estimated SD of 0.8, a minimum sample size of 11 centers was required. There is no prior publication of the analysis and comparison of subsection scores, rather only individual question means and SDs, so these are estimates by the authors.

### Qualitative Data Collection

We invited all actively participating US site primary investigators and any other relevant team members to be interviewed. We conducted semistructured phone interviews with site primary investigators at 8 US-based institutions (5 interviews site primary investigator only, 3 interviews site primary investigator plus at least 1 other team member). A single member of each research team (A.P.) conducted each interview using semistructured questions developed from the Consolidated Framework for Implementation Research (CFIR) qualitative interview guide (available at http://cfirstbank.com). The complete CFIR tool focuses on 5 domains^12^; however, we only included questions from the two contextual domains, outer setting and inner setting (**see Appendix II, Supplemental Digital Content 3,** http://links.lww.com/PQ9/A296 for full survey tool).^13,14^ We modified questions according to the research aims. We mapped these domains to the MUSIQ domains for consistency and ease of results interpretation.

### Qualitative Data Analysis

We conducted a thematic analysis of the transcribed interviews using both inductive and deductive analysis.^26^ Two qualitative researchers (A.P. and H.W.) worked independently and applied a priori codes based on an adapted version of the CFIR codebook. Next, we used emergent coding to identify additional themes not represented in the a priori codebook. We examined themes within the a priori defined subgroups, low and high implementers, and the low implementer subgroups of focused-approach low implementers and distributed approach low implementers. The qualitative researchers were blinded to the implementation category of the institution throughout the interviews and coding process.

## RESULTS

Over the 12 months before the completion of the MUSIQ tool and semistructured interviews, 7 of the 13 sites implemented 0–1 of the QI interventions recommended by the collaborative, categorizing them as low implementers, with 4 sites specifically identified as distributed approach low implementers and 3 sites focused-approach low implementers. The remaining 6 sites implemented at least 2 of 3 recommended QI interventions, categorizing them as high implementers (Table 1).

**Table 1.** Definitions of Successful Implementation of Recommended QI Interventions and the Number of Centers Who Met Criteria

QI Bundle Element	Definition of Successful Implementation	Centers
Rolling refreshers	Rolling refreshers completed for at least 50% of the high-risk patients	4
Hot debriefing	At least 50% of in-hospital cardiac arrests have a completed hot debrief.	7
Cold debriefing	If < 10 in-hospital cardiac arrests per year, cold debrief > 50% of events If > 10 in-hospital cardiac arrests per year, >10 cold debriefs per year	6

### Quantitative Assessment with MUSIQ Tool

**Fig. 1.**
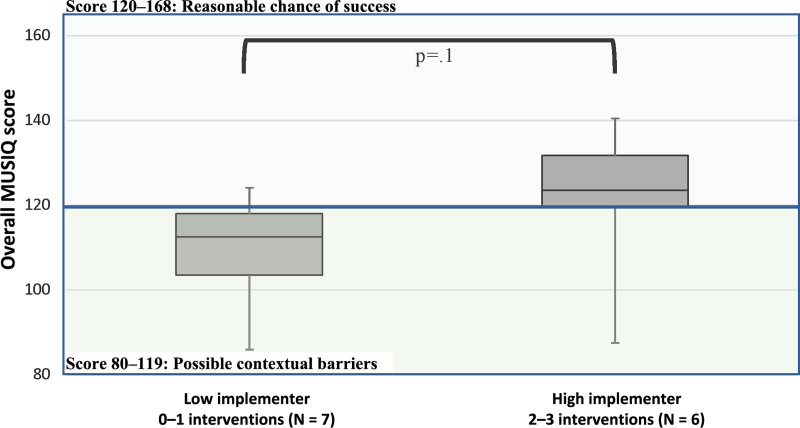
Comparison of median Total MUSIQ score for high (2–3 interventions) and low (0–1 intervention) implementers. Median score for low implementers was 112.6 and for high implementers was 123.6. For low implementers, 86.0 = min, 103.6 = 25th percentile, 112.6 = median, 119.6 = 75th percentile, and 124.2 = max. For high implementers, 87.6 = min, 119.6 = 25th percentile, 123.6 = median, 131.8 = 75th quartile, and 140.5 = max. The blue box indicates a score of 120–168 determined to have a reasonable chance of success, and the green box indicates a total score of 80–119, indicating possible contextual barriers.

All 13 actively participating US sites completed the MUSIQ questionnaire. Total MUSIQ scores ranged from 86.0 to 140.5 with a median of 118.7 and an IQR of 103.6–124.5 (Fig. 1). The median score for high implementers was 123.6 (IQR 119.6–132) and 112.6 for low implementers (IQR 103.6–118), *P* = 0.1. The majority of high implementers (66.7%) had a total score > 120, indicating a reasonable chance of success instead of only 28.6% of low implementers. This difference was not statistically significant. We evaluated the 6 subsection scores comparing low and high implementers. Evaluation of the QI team subsection noted a statistically significant difference in the mean score of 5.5 for low implementers and 6.1 for high implementers (*P* = 0.02). The mean subsection score for the external environment, organization, QI support and capacity, and microsystem was higher for high implementers than low implementers, although there were no statistically significant differences (Table 2).

**Table 2.** Mean Subsection Scores of MUSIQ Tool Compared via t-test, 1 = Totally Disagree and 7 = Totally Agree

MUSIQ Domain	Definition	Low Implementors (N = 7) Mean ± SD	High Implementors (N = 6) Mean ± SD	*P*
External environment	Community and society surrounding the organization	3.2 ± 1.4	3.6 ± 0.4	0.4
Organization	Largest collective unit that provides service to a population of patients	4.6 ± 0.7	5.3 ± 1.7	0.4
QI support and capacity	A system including financial support, data infrastructure, and workforce training to support QI work	3.1 ± 1.3	4.0 ± 1.5	0.3
QI Team	Group of individuals that work together on the QI project. The team is defined by their shared goals and mutual accountability for the QI project outcome	5.5 ± 0.4	6.1 ± 0.3	0.02
Microsystem	Small group of people working together on a regular basis to provide care to discrete populations of patients	5.0 ± 1.4	5.5 ± 1.5	0.5
Miscellaneous	Includes alignment with strategic goals and presence of a recent triggering event	2.2 ± 1.0	2.0 ± 0.9	0.7

### Qualitative Assessment with CFIR

Eight interviews with the local QI team leadership were conducted following the completion of the MUSIQ assessment. Of the 8 sites interviewed, 2 were distributive-approach low implementers, 3 as high implementers, and 3 focused-approach low implementers. Of the 5 sites that were not interviewed, 2 were distributive-approach low implementers and the remainder high implementers. There was no significant difference between responders and nonresponders in terms of months participating in the collaborative or the number of cardiac arrests entered into the dataset (**see Appendix III, Supplemental Digital Content 4,** http://links.lww.com/PQ9/A297). The reason most commonly given for declining a qualitative interview was time availability by the physician leader. The themes and quotes were similar between high implementers and focused-approach low implementers and differed from distributed approach low implementers.

*Contextual facilitators* as highlighted by high/focused low implementers and distributive-approach low implementers (Table 3).

**Table 3.** Qualitative Interview Results Mapped to MUSIC and CFIR Domains with Identification of Facilitators and Barriers with Representative Quotes

MUSIQ Theme		CFIR	Facilitator /Barrier	Representative Quotes
QI support and capacity	Resources/data infrastructure	Structural characteristics	Barrier 2: lack of resources	I’m pretty sure that we won’t have the resources in terms of data analytics, in terms of supply help, in terms of educational platform. I’m pretty sure none of those things are going to come through. So you have to have clever workarounds to get anything done if those are needed. (Center 6—High Implementor)
	Workforce focus on QI	Implementation climate	Barrier 4: lack of support	It’s not for a lack of interest. And it’s not for a lack of collaboration. There’s just not enough people… (Center 2—Low Distributed Approach Implementor)
				There’s leaders in place, there’s a conference in place, there’s pathways in place, but the actual on-the-ground work sometimes can be challenging. It’s hard to move the ball forward. (Center 5—High Implementor)
Organization	Culture	Culture	Barrier 4: lack of support	I felt like we, our leadership wasn’t interested in hearing it, and they wouldn’t have wanted us to start [getting feedback regarding a QI project]. (Center 8—Low Distributed Approach Implementor)
			Facilitator 2: failing forward culture	It’s okay to take risks, and it’s okay to make mistakes. (Center 7—Low Concentrated Approach Implementor)
	QI maturity	Structural characteristics	Facilitator 1: systems approach to QI	What used to be a very internal process, that something, say, that was held within the unit, is now viewed in a more systematic way across the whole institution… we have expanded the vision of what the scope of how problems are shared between areas instead of just each area doing their own thing…I think the idea of having more shared information is a good idea. It does mean that, at the local level, there can be confusion about who owns fixing a problem. (Center 6—High Implementor)
				There are structures in place that serve as vehicles for QI. So these are these initiatives that are specifically looking to target quality improvement, with the focus on patient safety, that have evidence behind it, leverage of science and safety, types of theories, and conception models, and then also has mechanisms that engage senior leadership to overcome some barriers that traditionally happen at the implementation side. (Center 7—Low Concentrated Approach Implementor)
				I think certain parts of the institution are well supported to do QI work. (Center 2—Low Distributed Approach Implementor)
	QI workforce	Readiness for implementation	Barrier 3: lack of formal QI training	No. I know that we have people, staff who work in the Safety and Quality Department but nobody that trains specifically on QI. (Center 8—Low Distributed Approach Implementor)
	Leadership		Barrier 4: lack of support	I think they’re [leaders] very disconnected. I think that’s one of the problems that we probably have. I think the organization doesn’t know what the unit needs and is thinking. And I don’t think that we’re, the unit knows and understands what the organization is thinking. (Center 5—High Implementor)
			Facilitator 3: leadership support	I think there’s, you know, at every level of leadership, there’s embracement of it. And I think that makes, that enables a culture, in general, of QI. So knowing that that’s, that people are expecting that at that level. (Center 4 – High Implementor)
External environ	External motivators	Peer pressure	Facilitator 5: knowledge of other institutions participating in QI	I think that it would make them more likely to buy in, especially since we had the site visits. And they saw that, you know, they saw that this was something that was important and people were coming and experts were coming to give their opinions. I think that made people more willing to listen. (Center 8—Low Distributed Approach Implementor)
Microsystem	QI culture	Culture	Facilitator 4: strong microculture and clear motivation	So we are given a lot of freedom and really encouraged to solve micro problems on our own. There’s huge emphasis that you pick something very, very small and make it better. And the converse of that is that we don’t really want you to think about the big picture items…that’s not really your role here. (Center 6—High Implementor)
				So in our unit, we actually have a really, really good culture. I think it’s hard to replicate. We have been very fortunate with how our unit supports itself. I would say the administration is, I mean, it’s as collaborative as you can expect an administration to be. I think they’re reasonable at listening, good at listening, but they can only support so much financially. (Center 2—Low Distributed Approach Implementor)
	Motivation			Sometimes, the outcome is a little more open-ended, in which case, it’s a little more entrepreneurial. Whereas, if they needed to, have us start an outcome, go make it hierarchal. And then if it’s urgent, like our bed management currently, then it’s rational, right, so it depends on the desired outcome and the timeline needed. (Center 2—Low Distributed Approach Implementor)
	Leadership	Readiness for implementation	Facilitator 3: leadership support	[QI] allows us to measure the quality of the care and ensure that we are, in a sustainable fashion, continuing to improve at giving [patients] the best care that we can. (Center 5—High Implementor)
				Our division director, so as a division, we have to set up certain goals, or [the division director] sets up certain goals for us to meet as to, in order to get incentives. And then if we meet those goals, then we get, basically, like a bonus or extra money because of it. (Center 8—Low Distributed Approach Implementor)
				if you’re very self-motivated and don’t need a lot of resources and are not making trouble, they’re not going to get in your business. And then if you either need their support and/or need resources and are, you know, make an effective argument, they are there for you. (Center 3—Low Focused-approach Implementor)
QI team	QI team leadership	Readiness for implementation	Facilitator 3: leadership support	So a lot of…doing that is the on the ground like data entry, collecting data, getting people to participate. That part I do largely on my own, and through time, have won over the support of a nurse educator who helps me…I think one of the struggles is that everyone, including the people on the data…are also double-stretched, and there’s not a lot of time to dedicate to it. And so they really have to pick what their priorities are, and their priorities are set by what the institution feels it wants to set, I guess. (Center 5—High Implementor)
				If it’s a simple, local project, it actually doesn’t ever go up to administration, because it’s done locally. And they give us a quality manager, analytical support in…and then your multidisciplinary team. So if you can do it locally within that team, it’s why they created the teams this way, it doesn’t ever actually escalate. So you approach the quality project that you want to approach within a given unit for any given year, and it’s not regulated from administration. (Center 2—Low Distributed-approach Implementor)
	QI Team Decision-Making Process	Implementation climate	Facilitator 6: clear prioritization of goals	I would say that in some ways, I think that we don’t have the resources that other institutions have…And so like some of the QI efforts that pedi-rescue has been doing, if we had had a lot of extra nurse educators or nurse researchers or, you know, nurses with extra time, we would have done some of the [collaborative] QI-focused things, and we have not yet. And so I think from that perspective, we are slow and measured and pick the ones that we think have the most evidence, or the ones, you know, of which we are most enamored. Center 3—Low Focused-approach Implementor)
				We rank every project based on its impact and its feasibility to make sure that we can actually implement things. And then we implement things on the side anyway, just because the unit wanted them, regardless of whether or not it was considered to be high impact or high yield, but it was something that we felt was pressing. So we will prioritize projects above kind of the standard prioritization, depending on how much we feel like we want this, and how much we’re invested in it. (Center 2—Low Distributed Approach Implementor)
	QI team tenure	Networks and communication	Barrier 1: low team tenure	Just the academic medicine flow in general. So it’s like, you know, incoming residents, incoming fellows, people that are, fully engaged leaving, so some of that sort of organizational or institutional knowledge and memory you have to work harder to keep up. (Center 3—Low Focused-approach Implementor)
Misc	Task strategic importance	Implementation climate	Barrier 4: lack of support	They do [set goals] but we’re not as metric minded as we should be. And it would be hard for me to even remember a goal that we had set. (Center 3—Low Focused-approach Implementor)
			Facilitator 3: leadership support	…there’s a target set for each year and a, for a lot of our QI stuff. A monitoring system to make, to see where we are as far as that target is concerned. (Center 5—High Implementor)

#### Facilitator Theme 1. Unified Institutional Approach to QI

High implementers and focused-approach low implementers identified a unified institutional approach or a standardized structure supported by their institution to improve the quality of care and provide resources as clear facilitators.

#### Facilitator Theme 2. A Fail Forward Climate

Focused-approach low implementers and high implementers mentioned that if something did not work, they were encouraged to try something new, demonstrating a fail forward^27^ implementation climate. Fail forward means to learn from failures or mistakes and apply that learning when moving forward in improvement.

#### Facilitator Theme 3. Leadership Support

Leadership support for QI at the microsystem, QI team, and organizational levels were all identified as facilitators for successful implementation. High implementers stated that receiving support beyond their division was a facilitator for success.

#### Facilitator Theme 4. Strong Microculture with Clear Motivation

A strong microsystem culture^14,28^ emphasizes teamwork, communication, and commitment to improving with clear motivation was identified as a facilitator by all groups.

#### Facilitator Theme 5. Knowledge of Other Organizations Participating in QI

All groups found sharing with key stakeholders that other institutions were participating in this collaborative improved support. Interviewees explicitly mentioned site visits as a facilitator to improving support.

#### Facilitator Theme 6. Prioritization of Goals

A clear prioritization of goals within a QI team was a facilitator for success identified by all groups.

*Contextual barriers* as identified by all sites (Table 3).

#### Barrier Theme 1. Low Team Tenure

All sites identified low team tenure, including rotating medical providers such as residents and fellows, as a barrier to the successful implementation of the QI resuscitation bundle by all.

#### Barrier Theme 2. No Specific Allocation of Resources or Time for QI

High and both groups of low implementers identified a lack of resources and no specific time allocation for QI for staff as primary barriers.

#### Barrier Theme 3. Lack of Formalized QI Knowledge or Training

Sites identified as distributed approach low implementers mentioned a lack of formal QI training for themselves or their team members as a barrier.

#### Barrier Theme 4. Lack of Support and Buy-in by Leaders and Staff

Lack of support and buy-in was mentioned by all groups as a significant barrier to implementation. Whether the participant felt that they had institutional buy-in or not, each institution mentioned working individually to gain champions and stakeholders’ buy-in and the difficulties associated with getting the work done “on the ground.”

## DISCUSSION

This study describes the critical contextual factors as determined by the local site leader associated with successfully implementing a multicenter collaborative resuscitation QI bundle using mixed methods. To facilitate the dissemination of improvement interventions, knowledge of contextual factors is necessary.^29^ However, the influence of contextual factors is poorly reported in the literature.^30^ The quantitative data derived from the MUSIQ tool demonstrates higher contextual scores for all components in those centers that successfully implemented 2–3 interventions. We found that only the local QI team’s strength was statistically associated with the successful implementation of QI bundle elements with substantial variation between centers. The other MUSIQ domains of the external environment, organization, QI support and capacity, and microsystem all scored higher, that is, meaning more capacity and support for high implementers. However, due to the small sample size and variation in responses among centers, the score difference between high and low implementers was not significant. Through qualitative interviews, we identified specific contextual facilitators and barriers that allowed for future interventions to improve compliance. Contextual facilitators included institutional-wide support of QI, a failing forward culture, leadership support, a strong microculture with clear motivation, and prioritization of goals. High and low implementers experienced similar barriers with low distributed approach implementers citing a lack of QI knowledge and experience as a specific barrier, reinforcing the importance of a well-trained and robust QI team as seen in our MUSIQ results and prior research around contextual factors.^30,31^ Both high and low implementers found that an overall lack of support and buy-in from staff and leadership served as substantial barriers.

Although the contextual facilitators and barriers identified are unlikely to be surprising to QI leaders, a better understanding of the contextual effects on intervention success can help local centers advocate for necessary resources. QI research must focus not just on the effects of the intervention but also on the contextual factors that influence improvement.^13,32^ As large multicenter QI collaboratives continue to grow, it is incumbent on the leaders of collaboratives to understand the importance of context on individual sites’ success.^13^ Tools including the quantitative MUSIQ calculator and the qualitative CFIR guide provide an opportunity for sites to evaluate the impact of context on success of QI initiatives.^14,33–35^ Site-specific evaluation is vital to the future success of QI multicenter collaboratives as a focus on context helps to elucidate some possible explanations for differences in the implementation of recommended interventions. We would recommend that contextual tools, like MUSIQ and CFIR, be used before initiating centers within quality collaboratives to assess the contextual facilitators and barriers at individual sites. With these data, leaders can develop custom intervention implementation bundles in which resources, mentorship, and other support are tailored to their site’s specific contextual needs.

Our study has multiple limitations. First, the physician site leader completed a single MUSIQ tool at each of the 13 actively participating US sites. It is possible that this approach failed to capture a full picture of the local context by not taking a more multidisciplinary approach to completion. Also, these data were self-reported by the site leader and were not independently verified in any way by the study team. As our focus was on the facilitators and barriers experienced by the local site leader in implementation for the collaborative, we chose not to survey other team members. Second, our qualitative interviews were single interviews at one point in time and may not accurately represent the changes in contextual factors that have occurred over time since the sites joined the collaborative at various time points.

Furthermore, as most interviews only included the site primary investigators and were conducted with a subset of the group, selection bias may have been present. Although there were no significant differences in the months of participation, cardiac arrests enrolled, or proportion of low implementers in the nonresponders, it is possible that the omission of these 5 centers impacted the conclusions of the qualitative results. Last, we did not have adequate power to assess the MUSIQ subscore differences between distributed approach low implementers and focused-approach low implementers. Qualitative results suggest that focused-approach low implementers may be more similar to high implementers in facilitators and barriers. This hypothesis-generating finding provides a foundation for future, more extensive studies examining context.

## CONCLUSIONS

Using mixed methods, we showed an association between the local QI team’s strength and the ability to implement recommended resuscitation QI interventions while further identifying facilitators and barriers to implementation. These data support the importance of a well-trained and influential QI team to successfully implement QI initiatives. Furthermore, successful implementation of QI initiatives within a large collaborative requires an understanding of each institution’s context-specific framework. Local leadership engagement, available resources, and access to knowledge may help sites to be successful.

## DISCLOSURE

The authors have no financial interest to declare in relation to the content of this article.

Pediatric Resuscitation Quality (pediRES-Q) Collaborative Investigators: Diane Atkins, University of Iowa Stead Family Children’s Hospital, Iowa City, Iowa; Shilpa Balikai, University of Iowa Stead Family Children’s Hospital, Iowa City, Iowa; Marc Berg, Lucile Packard Children’s Hospital, Palo Alto, Calif.; Robert Berg, The Children’s Hospital of Philadelphia, Philadelphia, Pa.; Matthew S. Braga, Dartmouth-Hitchcock Medical Center, Lebanon, N.H.; Corinne Buysse, Erasmus MC–Sophia Children’s Hospital, Rotterdam, The Netherlands; Allan DeCaen, Stollery Children’s Hospital, Edmonton, AB, Canada; Jimena del Castillo, Hospital Maternoinfantil Gregorio Marañón, Madrid, Spain; Aaron Donoghue, The Children’s Hospital of Philadelphia, Philadelphia, Pa.; Jordan Duvall-Arnold, Johns Hopkins University School of Medicine, Baltimore, Md.; Ivie Esangbedo, UT Southwestern Medical Center, Dallas, Tex.; Stuart Friess, St. Louis Children’s Hospital, St. Louis, Mo.; Janie Garza, Medical City Children’s Hospital, Dallas, Tex.; Elaine Gilfoyle, Alberta Children’s Hospital, Calgary, AB, Canada; Sarah Haskell, University of Iowa Stead Family Children’s Hospital, Iowa City, Iowa; Betsy Hunt, Johns Hopkins University School of Medicine, Baltimore, Md.; Takanari Ikeyama, Aichi Children’s Health and Medical Center, Ohbu, Aichi Prefecture, Japan; Dean Jarvis, Dartmouth-Hitchcock Medical Center, Lebanon, N.H.; David Kessler, New York-Presbyterian Morgan Stanley Children’s Hospital, New York, N.Y.; Lynda Knight, Lucile Packard Children’s Hospital, Palo Alto, Calif.; Hiroshi Kurosawa, Kobe Children’s Hospital, Hyōgo Prefecture, Japan; Dori-Ann Martin, Alberta Children’s Hospital, Calgary, AB, Canada; Michael Meyer, Children’s Hospital of Wisconsin, Milwaukee, Wis.; Yee Hui Mok, KK Women’s & Children’s Hospital, Singapore; Ryan Morgan, The Children’s Hospital of Philadelphia, Philadelphia, Pa.; Sholeen Nett, Dartmouth-Hitchcock Medical Center, Lebanon, N.H.; Dana E. Niles, The Children’s Hospital of Philadelphia, Philadelphia, Pa.; Gene Ong, KK Women’s & Children’s Hospital, Singapore; Jacqueline Ong, National University Children’s Medical Institute, Singapore; Tara Petersen, Children’s Hospital of Wisconsin, Milwaukee, Wis.; Robyn Puente, UT Southwestern Medical Center, Dallas, Tex.; Prakad Rajapreyar, Children’s Hospital of Wisconsin, Milwaukee, Wis.; Lindsay Ryerson, Stollery Children’s Hospital, Edmonton, AB, Canada; Barney Scholefield, Birmingham Children’s Hospital, Birmingham, United Kingdom; Anita Sen, New York-Presbyterian Morgan Stanley Children’s Hospital, New York, N.Y.; Roopa Seshadri, The Children’s Hospital of Philadelphia, Philadelphia, Pa.; Yuko Shiima, Kobe Children’s Hospital, Hyōgo Prefecture, Japan; Naoki Shimizu, Tokyo Metropolitan Children’s Medical Center, Tokyo, Japan; Marcy Singleton, Dartmouth-Hitchcock Medical Center, Lebanon, N.H.; Felice Su, Lucile Packard Children’s Hospital, Palo Alto, Calif.; Robert M. Sutton, The Children’s Hospital of Philadelphia, Philadelphia, Pa.; Todd Sweberg, Cohen Children’s Medical Center, New Hyde Park, N.Y.; Javier Urbano Villaescusa, Hospital Maternoinfantil Gregorio Marañón, Madrid, Spain; Denise Welsby, Great Ormond Street Hospital, London, United Kingdom; Andrea Yeo, National University Children’s Medical Institute, Singapore; Pricilla Yu, UT Southwestern Medical Center, Dallas, Tex.

## ACKNOWLEDGMENTS

The authors thank Dr. Heather Kaplan for providing the Model for Understanding Success in Quality (MUSIQ) survey for this study.

## Supplementary Material


