# Supplementary material for: Contextual Factors Affecting Implementation of In-hospital Pediatric CPR Quality Improvement Interventions in a Resuscitation Collaborative
Source: Pediatr Qual Saf. 2021 Aug 26;6(5):e455. doi: 10.1097/pq9.0000000000000455 (PMC8389879; doi:10.1097/pq9.0000000000000455)
Supplement: Supplementary file 1 [file pqs-6-e455-s001.pdf]

Good Reporting of A Mixed Methods Study (GRAMMS) Checklist

Guideline	Section: Page
Justification to use a mixed methods approach to the research question	Materials and Methods: p. 6
Articulation of the design in terms of purpose, priority, and sequence of methods	Materials and Methods: pp.6-10
Describe each method in terms of sampling, data collection and analysis	Materials and Methods: Quantitative Data Collection & Quantitative Data Analysis: pp.8-9<br><br>Qualitative Data Collection & Qualitative Data Analysis: pp.9-10
Delineate where and how integration occurs and who has participated in it	Materials and Methods: pp. 9-10<br><br>Discussion: p.14
Describe any limitation of one method associated with the presence of another	Discussion: p.15
Describe insights gained from mixing or integrating methods	Discussion: pp.13-14

O'Cathain A, Murphy E, Nicholl J. The quality of mixed methods studies in health services research. J Health Serv Res Policy. 2008;13: 92-98.
