# Supplementary material for: Contextual Factors Affecting Implementation of In-hospital Pediatric CPR Quality Improvement Interventions in a Resuscitation Collaborative
Source: Pediatr Qual Saf. 2021 Aug 26;6(5):e455. doi: 10.1097/pq9.0000000000000455 (PMC8389879; doi:10.1097/pq9.0000000000000455)
Supplement: Supplementary file 2 [file pqs-6-e455-s002.pdf]

Appendix I: Description of QI Interventions. Further description can be found here: https://www.pedires-q.org/qi-bundle

QI Intervention	Description
**Rolling Refresher**	A designated clinician briefs a bedside provider for possible challenges of care, optimal positioning of the patient during an emergency event, and refreshing of psychomotor skills via a portable manikin/defibrillator system with chest compression sensor provide automated corrective feedback to optimize CPR skills.
**Hot Debrief**	Brief (5 minute) immediate post-event feedback, discussion, and emotional processing of arrest event.
**Cold Debrief**	Multi-disciplinary event review utilizing event and patient data to discuss process of care and team performance.
