# Supplementary material for: Contextual Factors Affecting Implementation of In-hospital Pediatric CPR Quality Improvement Interventions in a Resuscitation Collaborative
Source: Pediatr Qual Saf. 2021 Aug 26;6(5):e455. doi: 10.1097/pq9.0000000000000455 (PMC8389879; doi:10.1097/pq9.0000000000000455)
Supplement: Supplementary file 3 [file pqs-6-e455-s003.pdf]

Appendix II: Qualitative Interview Script

To modify the CFIR interview guide (MD, HW, and AP), we compared the components that make up the 5 domains of the CFIR tool to the components that make up the 6 domains of MUSIQ tool. We first eliminated those domains that were not considered aspects of context from both tools. Then we independently reviewed the remaining components that existed in both the MUSIQ domains and the CFIR. Using a consensus mapping method we mapped the components of the MUSIQ tool that related to context to the two context related domains of the CFIR. Based on this process we developed a modified interview guide focused on contextual factors that influence implementation of QI.

Thank you for taking the time to talk with me today. I am here to talk with you about the role of context and environment on Quality Improvement success. The questions that I ask will help elucidate barriers and facilitators to implementation of a resuscitation bundle within your institution. Before we get started are you okay with this interview being audio recorded?
Do you have any questions for me before we begin?

Inner Setting

First we will talk about the **Structural Characteristics** of your institution. This can include the social architecture, age, maturity, size, or physical layout.

1. How does the infrastructure of your organization affect the implementation of QI interventions?
    - (PROBE: What is an example of how the infrastructure **facilitates/hinders** implementation of QI interventions?)
    - (PROBE: How do you work around structural challenges?)

The next few questions will focus on **Culture.** In this context culture is defined as the general beliefs, values, assumptions that people embrace)

1. How would you describe the culture of your organization?
    - Do you feel like the culture of your own unit is different from the overall organization? In what ways?
2. How do you think your organization's culture affects the implementation of QI?
    - (PROBE: Can you describe an example that highlights this?)
3. (PROBE: Do you think your organization is receptive to implementing QI interventions?)
    - (PROBE: Why do you think that is?)
4. To what extent are new ideas embraced and used to make improvements in your organization?
    - (PROBE: Can you describe a recent example?)
5. Some people characterize culture in terms of four general types. To what extent would you characterize your culture as:
    - Team (Clan) Culture (Flexible, Internal Focus): A friendly workplace where leaders act like mentors, facilitators, and team-builders. There is value placed on long-term development and doing things together.
    - Hierarchical (Hierarchy) Culture (Control, Internal Focus): A structured and formalized workplace where leaders act like coordinators, monitors, and organizers. There is value placed on incremental change and doing things right.
    - Entrepreneurial (Adhocracy) Culture (Flexible, External Focus): A dynamic workplace with leaders that stimulate intervention. There is value placed on breakthroughs and doing things first.
    - Rational (Market) Culture (Control, External Focus): A competitive workplace with leaders like hard drivers, producers, or competitors. There is value placed on short-term performance and doing things fast.

(PROBE: Can you explain the percentages that you gave?)

Compatibility

1. How well does QI fit with your values and norms ?
2. What about with the values and norms within the organization?
3. How is QI integrated into current processes?
   - How does it interact or conflict with current programs or processes?
   - Can you think of an example of how QI is integrated into current processes?

Relative Priority

1. What are the activities or initiatives that appear to have highest priority for the organization.
2. How does the priority of implementing QI interventions compare to other priorities in your organization?
   - (PROBE: Do you think QI initiatives take a backseat to other high-priority initiatives going on now?)

Goals & Feedback

1. Does your organization/unit set goals related to the implementation of QI interventions?
   1. (PROBE: How are goals communicated in the organization? To whom are they communicated?)
   2. (PROBE: Are changes made based on how things are going? Can you give an example?)
2. How does implementation of QI interventions align with other organizational goals?
3. Are employee evaluations or employee advancement connected to or influenced by involvement in QI implementation?

Learning Climate

1. To what extent do you feel like you can try new things to improve your work processes?
   - What role, if any, did your supervisor (or other leaders) play? What actions did they take?

Leadership Engagement

1. What level of endorsement or support have you seen or heard from leaders?
2. What level of involvement has leadership at your organization had so far with QI interventions?

Available Resources

1. Do you expect to have sufficient resources to implement and administer QI interventions?
   - [If Yes] What resources are you counting on? Are there any other resources that you received, or would have liked to receive?
   - What resources will be easy to procure?
   - [If no] What resources will not be available?

The next few questions will focus more on the Process of implementing QI interventions.

Planning

1. Tell me about putting a plan in place to implement QI interventions. What are the steps that you normally take?

- (PROBE: Who is involved in the planning process? What are their roles?)
- (PROBE: Are the appropriate people involved in the planning process? How engaged are they?)

Now we are going to talk about 4 key roles: Opinion leaders, champions, external change agents, and key stakeholders. Top down:

Opinion Leaders – Those who have representative opinions of your group and who people look to for opinion. Good examples are experienced nurses who are in leadership roles, physician medical directors of a unit, or nurses or respiratory therapist who serve in chare role.

1. Who are the key influential individuals to get on board with implementation efforts?
    - How do you think these people will influence others' engagement with QI interventions? The success of the implementation?

Champions - They are often the leader of a project or QI intervention. Work to drive their own QI interventions as well as other peoples' QI interventions.

1. Other than the formal implementation leader, are there people in your organization who are champions of QI interventions?
    - Were they formally appointed in this position, or was it an informal role?
    - What position do these champions have in your organization?

External Change Agents

1. Does someone (or a team) outside your organization help you with implementing QI interventions?
    - What is their role?
    - What kind of activities will they be doing?
    - How helpful do you think he/she/they will be? In what ways?

Key Stakeholders – any party who may have an interest in the QI actions or will be affected by its outcomes. Those with the political or professional clout to make things happen. Those affected by the changes that should have a voice in the process.

1. Who are the key stakeholders of QI interventions?
    - What steps to you take to encourage individuals to commit to using QI interventions?
    - What information do you give them?
    - How frequently and how do you communicate with them?

Outer Setting

Peer Pressure

1. What do you know about other organizations that have implemented QI interventions?
    - How does this affect support for implementing QI interventions in your setting?
2. To what extent does/would implementing QI interventions provide an advantage for your organization compared to other organizations in your area?
