# Supplementary material for: Contextual Factors Affecting Implementation of In-hospital Pediatric CPR Quality Improvement Interventions in a Resuscitation Collaborative
Source: Pediatr Qual Saf. 2021 Aug 26;6(5):e455. doi: 10.1097/pq9.0000000000000455 (PMC8389879; doi:10.1097/pq9.0000000000000455)
Supplement: Supplementary file 4 [file pqs-6-e455-s004.pdf]

Appendix III: Responders Versus Non Responders for Qualitative Interviews

	Responders (N=8)	Non-responders (N=5)	p-value
Low Implementers, % (N)	25% (2)	40% (2)	0.6
Mean Months in Collaborative ± SD	25.1 ± 6.5	31 ± 0.7	0.08
Mean Number of Cardiac Arrests Enrolled ± SD	38.6 ± 19.0	28.0 ± 26.2	0.4
